# A unified computational model for revealing and predicting subtle subtypes of cancers

**DOI:** 10.1186/1471-2105-13-70

**Published:** 2012-05-01

**Authors:** Xianwen Ren, Yong Wang, Jiguang Wang, Xiang-Sun Zhang

**Affiliations:** 1 MOH Key Laboratory of Systems Biology of Pathogens, Institute of Pathogen Biology, Chinese Academy of Medical Sciences and Peking Union Medical College, Beijing 100730, China; 2 Academy of Mathematics and Systems Science, Chinese Academy of Sciences, Beijing 100190, China; 3 National Center for Mathematics and Interdisciplinary Sciences, Chinese Academy of Sciences, Beijing 100190, China; 4 Beijing Institute of Genomics, Chinese Academy of Sciences, 7 Beitucheng West Road, Beijing 100029, China

**Keywords:** Class discovery, Class prediction, Quadratic programming, Cancer

## Abstract

**Background:**

Gene expression profiling technologies have gradually become a community standard tool for clinical applications. For example, gene expression data has been analyzed to reveal novel disease subtypes (class discovery) and assign particular samples to well-defined classes (class prediction). In the past decade, many effective methods have been proposed for individual applications. However, there is still a pressing need for a unified framework that can reveal the complicated relationships between samples.

**Results:**

We propose a novel convex optimization model to perform class discovery and class prediction in a unified framework. An efficient algorithm is designed and software named OTCC (Optimization Tool for Clustering and Classification) is developed. Comparison in a simulated dataset shows that our method outperforms the existing methods. We then applied OTCC to acute leukemia and breast cancer datasets. The results demonstrate that our method not only can reveal the subtle structures underlying those cancer gene expression data but also can accurately predict the class labels of unknown cancer samples. Therefore, our method holds the promise to identify novel cancer subtypes and improve diagnosis.

**Conclusions:**

We propose a unified computational framework for class discovery and class prediction to facilitate the discovery and prediction of subtle subtypes of cancers. Our method can be generally applied to multiple types of measurements, e.g., gene expression profiling, proteomic measuring, and recent next-generation sequencing, since it only requires the similarities among samples as input.

## Background

Accurate diagnosis is a great challenge for clinical therapies. In particular, the current diagnosis based on only a few genes, proteins or metabolites are very limited when it comes to tackling the intrinsic complexity of many diseases, e.g., cancers. Fortunately with the rapid development of high-throughput technologies, gene expression profiling techniques have been widely applied in clinical research. The big advantage is to simultaneously measure the expressions of thousands of genes [1-4]. To date, two types of strategies have been widely used to analyze gene expression data for clinical purpose: class discovery and class prediction. Class discovery tries to identify new disease subtypes while class prediction tries to assign particular samples to well-defined disease classes [5]. Both tasks have significant potentials to improve cancer diagnosis, prognosis, and therapies but require effective and efficient computational methods to deal with the large amount of data involved.

In the machine learning framework, class discovery is an unsupervised task. Many methods related to clustering have been proposed and applied to identify new disease subtypes. Several well-known methods, e.g., hierarchical clustering (HC), self-organizing maps (SOM), and non-negative matrix factorization (NMF) have been successfully used [6-14]. Class prediction is generally supervised. Supervised methods, e.g., support vector machines, Bayes classifiers, *k* nearest neighbors, etc., have been adopted [15-19]. However, class discovery and class prediction are by nature closely linked to each other. Their separate computational implementation prevents clinicians from comparing the results obtained in unsupervised and supervised settings. Alexandridis et al. developed a mixture model unifying two tasks and obtained promising results [20]. However, the global optimum of their model cannot be guaranteed in theory and is difficult to obtain in practice. In addition, estimating the mixture distributions often involves profiling the gene expressions of many clinical samples, which is time consuming and also very expensive. Therefore, a universal, easily solvable computational framework is highly desirable to help clinicians understand such diseases using fewer samples.

In this paper, we propose a semi-supervised solution to formulate class discovery and class prediction into a unified framework. We term it OTCC (Optimization Tool for Clustering and Classification). The underlying principle is to seek an optimal sample labeling scheme to ensure that similar samples can be assigned with similar labels. This assumption is straightforward and can be easily understood by clinicians. OTCC has several prominent features: 1) The global optimal solution is guaranteed because it is based on convex quadratic programming; 2) It implements class discovery and class prediction in one computational framework; 3) It does not require many samples; 4) It can be applied to both small and large datasets due to a customized iterative algorithm. Experiments on acute leukemia and breast cancer datasets suggest the validity and advantages of OTCC in mining the clinical significance of patient gene expression data.

## Methods

### Overview of the optimization model

For simplicity, we consider two classes to illustrate the optimization model. We note that both class discovery and class prediction for the two classes can be transformed into a sample labeling problem. In this section, the optimization model is formulated to find the best way to assign labels to the samples. The labeling problem for multi-class cases for class discovery and class prediction will be discussed in the next sections.

For two-class cases, we denote one class by zero and the other class by one. Assume all the sample labels are continuous variables between zero and one. The objective of the optimization model is to assign similar labels to similar samples as much as possible. The formulations are given as follows:

(1) $$\min_{f}\frac{1}{2}\sum_{i=1}^{N}\sum_{j=1}^{N}s_{\mathrm{ij}}{\left(f_{i}-f_{j}\right)}^{2}$$

Subject to

(2) $$f_{a}=0(a\in A)\text{,}\;f_{b}=1(b\in B)\text{ and }0\leq f_{i}\leq 1(i\in \{1,\cdots ,N\})$$

where *N* is the total number of samples; *s*_*ij*_ is the similarity score of samples *x*_*i*_ and *x*_*j,*_ which is calculated from the gene expression profiles; and *f*_*i*_ is the unknown variable to be determined and represents the label of sample *x*_*i*_. *A* is a set of samples that are known to belong to Class Zero. *B* is a set of samples that are known to belong to Class One. The objective function in Equation (1) tends to assign similar labels to similar samples ($s_{\mathrm{ij}}>0$). Constraints in Equation (2) ensure that the resultant sample labels are consistent with the known information and that the final labels $f_{i}$ are between zero and one.

The objective function (1) can be rewritten in vector form as $f^{T}Lf$. Here *f* is the sample label vector (*f*_*i*,_ is the label of Sample *i*) and *L* is the Laplacian matrix of the similarity matrix *S* (*s*_*ij*_, the similarity score of samples *i* and *j*), i.e., *L* = *D* − *S* and *D* is a diagonal matrix with $d_{\mathrm{ii}}=\sum_{j=1}^{N}s_{\mathrm{ij}}$. If *s*_*ij*_ are all non-negative, *L* is positive semi-definite. The objective function is convex and the constraints are linear. Thus the model (1–2) is a convex quadratic programming problem and a global optimal solution is guaranteed.

Due to the form of the objective function, our optimization model is tightly related to spectral clustering and semi-supervised learning [21-23]. These links form the basis for class discovery and class prediction. Importantly, the constraints imposed in this model provide a few advantages for cutoff setting and outlier identification.

### The sample similarity matrix

Usually the gene expression profile for *n* genes and *m* samples is mathematically denoted by an n×m matrix X. Each element *x*_*ij*_ represents the expression level of gene *i* in sample *j*. *x*_*i*_ is an m-dimensional vector denoting the expression value of gene *i*. The construction of the sample similarity matrix is important because it is the only input for model (1–2) to fully utilize the gene expression data. Since the calculation of the similarity matrix and the solving of the optimization model are separated, various feature selection/extraction techniques and different measures of similarity can be applied here to incorporate prior information. A simple and straightforward method to construct a similarity matrix of samples based on the gene expression profiles is to calculate the Pearson correlation coefficients of each sample pair which provides a uniform measure between −1 and 1. To get non-negative *s*_*ij*_, a linear transformation can be adopted to map [−1, 1] to [0, 1]. Because the Pearson correlation coefficients based on the gene expression profiles are calculated pairwisely between every two samples, it does not consider the similarities among samples globally. To provide a global similarity measure, a second-order correlation similarity matrix can be constructed by exploiting the deduced sample correlation features (i.e., calculating the Pearson correlation coefficients of the sample correlation vectors). In this study we used second-order correlation similarity matrices to identify the underlying structures of cancer gene expression data.

### Setting for class discovery

Given the similarity matrix *S*, sets *A* and *B* are necessary to implement the class discovery task through Model (1–2). If *A* and *B* are not provided, i.e., without the corresponding constraints in Equation (2), the optimization model results in a trivial solution given non-negative *s*_*ij*_. The trivial solution indicates that all the samples belong to one class, which is meaningless. To obtain a meaningful solution, *A* and *B* should be specified and intersection between *A* and *B* is not allowed. Usually for class discovery task, information about *A* and *B* is not available since all sample labels are unknown. Here we introduce a weak assumption to set up *A* and *B*. We name it here as the most dissimilar assumption. The assumption is that the two least similar samples should belong to different classes. Otherwise all samples should belong to one class. According to this assumption, the minimal *s*_*ij*_ for $i,j\in \left\{1,\cdots ,N\right\}$ is identified, denoted by *s*_*ab*_. Let Sample *x*_*a*_ be labeled with zero and *x*_*b*_ be labeled with one, or vice versa. If there is more than one minimal value in S, the sample pair with minimal values in S^n^ (the power of similarity matrix S, where n > 1 is a positive integer) is also a candidate to determine set A and B. Model (1–2) is then well constructed and optimal labeling can be uniquely determined by solving the model.

### Setting for class prediction

Class prediction tries to assign a set of particular samples to known classes. In this setting, gold-standard data are generally available and some gene expression profiles for samples are labeled with known classes. That is, *A* and *B* are available. Model (1–2) can therefore be implemented for class prediction.

### A fast algorithm for large-scale problems

Model (1–2) can be considered convex quadratic programming if all values of *s*_*ij*_ are positive. It can be solved efficiently by the general solvers such as quadprog in Matlab and the sequential minimal optimization (SMO) algorithm which has been applied successfully to solve the optimization problems in support vector machine applications. Here, a simple customized algorithm is proposed to solve Model (1–2) quickly, even for very large-scale problems by fully considering its particular characteristics.

The Lagrange function of optimization model (1–2) is:

(3) $$\Psi =\frac{1}{2}\sum_{i=1}^{N}\sum_{j=1}^{N}s_{\mathrm{ij}}{\left(f_{i}-f_{j}\right)}^{2}+\sum_{a\in A}\alpha_{a}f_{a}+\sum_{b\in B}\beta_{b}(f_{b}-1)-\sum_{i=1}^{N}\mu_{i}f_{i}+\sum_{i=1}^{N}\nu_{i}(f_{i}-1)$$

Then the Karush-Kuhn-Tucker (KKT) conditions are:

(4) $$\mu_{i}-\nu_{i}=2\sum_{j=1}^{N}s_{\mathrm{ij}}(f_{i}-f_{j})\text{,}\;\mu_{i}f_{i}=0\text{,}\;\nu_{i}(f_{i}-1)=0\text{,}\;\mu_{i}\geq 0\text{, }\nu_{i}\geq 0\text{, }0\leq f_{i}\leq 1\;(i\in \{1,\cdots ,N\})$$

(5) $$f_{a}=0(a\in A)\text{ and }f_{b}=1(b\in B)$$

These conditions can be reduced as:

(6) fi=0 or fi=1 or fi=∑i=1Nsijfj∑j=1Nsij(i∈{1,⋯,N}, i∉A, i∉B),fa=0(a∈A) and  fb=1(b∈B)

We design the following algorithm to quickly find the solution:

Algorithm 1.

· **Step 1**: Let t=0 and $f_{a}=0$ for a∈A, $f_{b}=1$ for b∈B and $f_{i}=0$ for $i\in \left\{1,\cdots ,N\right\}/A/B$.

· **Step 2**: Calculate $f_{i}^{t+1}=\frac{\sum_{j=1}^{N}s_{\mathrm{ij}}f_{j}^{t}}{\sum_{j=1}^{N}s_{\mathrm{ij}}}$ for $i\in \left\{1,\cdots ,N\right\}/A/B$.

· **Step 3**: Let t=t+1. If $\max_{i}|f_{i}^{t}-f_{i}^{t-1}|$ is less than a predefined threshold or *t* is larger than the maximal steps allowed, stop; otherwise, repeat **Step 2** and **Step 3**.

Next, we prove the above algorithm is correct and convergent.

**Theroem 1:** Suppose **Algorithm 1** gives rise to the sequence,$f^{0},f^{1},\ldots ,f^{t},f^{t+1},\ldots$. It converges to $f^{*}$. $f^{*}$ satisfies the KKT point of Model (1)-(2).

Firstly, we prove that **Algorithm 1** is convergent. The Lagrangian function of our optimization model (1–2) is as follows,

(7) $$\Psi (f)=\frac{1}{2}\sum_{i=1}^{N}\sum_{j=1}^{N}s_{\mathrm{ij}}{\left(f_{i}-f_{j}\right)}^{2}+\sum_{a\in A}\alpha_{a}f_{a}+\sum_{b\in B}\beta_{b}(f_{b}-1)-\sum_{i=1}^{N}\mu_{i}f_{i}+\sum_{i=1}^{N}\nu_{i}(f_{i}-1)$$

Then an auxiliary function $\Phi (f,f^{'})$ is constructed for the Lagrangian function

(8) $$\Phi (f,f’)=\sum_{\mathrm{ij}}f_{i}^{,}L_{\mathrm{ij}}f_{j}^{,}(1+\log\frac{f_{i}f_{j}}{f_{i}^{,}f_{j}^{,}})+\sum_{a\in A}\alpha_{a}f_{a}+\sum_{b\in B}\beta_{b}(f_{b}-1)-\sum_{i=1}^{N}\mu_{i}f_{i}+\sum_{i=1}^{N}\nu_{i}(f_{i}-1)$$

where *L* is the Laplacian matrix of the similarity matrix *S*. The auxiliary function satisfies $\Phi (f,f^{'})\leq \Psi (f)\text{,}\;\Phi (f,f)=\Psi (f)$. The second order derivative of $\Phi (f,f^{'})$ with respect to  is calculated as

(9) $$\frac{\partial^{2}\Phi (f,f')}{\partial f_{i}\partial f_{j}}=-[2\frac{f{'}_{i}{\left(Lf'\right)}_{i}}{f_{i}^{2}}]\delta_{\mathrm{ij}}$$

where $\delta_{\mathrm{ij}}$is the Kronecker delta function, i.e., $\delta_{\mathrm{ij}}=1$ when i = j and $\delta_{\mathrm{ij}}=0$ otherwise. Since *L* is positive semi-definite, $\Phi (f,f^{'})$ is concave in *f*. We can obtain global maxima when the first order derivative is zero.

(10) $$\frac{\partial \Phi (f,f')}{\partial f_{i}}=2\frac{f{'}_{i}{\left(Lf'\right)}_{i}}{f_{i}}-u_{i}+v_{i}=0$$

Recalling the KKT condition and our iterative **Step 2** can be reformulated as,

(11) $$f^{t+1}=\arg\max_{f}\Phi (f,f^{t})$$

By the property of the auxiliary function, we have

(12) $$\Psi \left(f^{t}\;\right)=\Phi (f^{t},f^{t})\leq \Phi (f^{t+1},f^{t})\leq \Psi \left(f^{t+1}\;\right)$$

(13) $$\Psi \left(f^{0}\;\right)\leq \Psi \left(f^{1}\;\right)\leq L\cdots \Psi \left(f^{t}\;\right)\leq \Psi \left(f^{t+1}\;\right)\leq L\cdots$$

Ψ(f) is monotonically increasing and is bounded from above. Thus our algorithm converges.

Secondly we show **Algorithm 1** is correct. At convergence, the solution is $f^{*}$ and satisfies $f_{i}^{*}=\frac{\sum_{j=1}^{N}s_{\mathrm{ij}}f_{j}^{*}}{\sum_{j=1}^{N}s_{\mathrm{ij}}}$ for i∈{1,⋯,N}/A/B. $f_{a}^{*}=0$for a∈A and $f_{b}^{*}=1$ for b∈Balso hold. Then $f^{*}$ satisfies the KKT condition (4)-(5). This proves our algorithm correctly converges to a minimum satisfying KKT condition.

One advantage of our algorithm is that the computational complexity is low and it requires only a small amount of computer memory. So our algorithm can be applied to very large data sets.

### Post-processing the solutions

Each sample gets a continuous label between zero and one after the optimization model (1)-(2) is solved. We can easily obtain the binary labels by applying a pre-defined threshold. If a training data set is available, this threshold can be learned from the training data by cross-validation. Otherwise, the median of zero and one, 0.5, is a natural cutoff to convert the continuous labels into binary labels. If label $f_{i}$ is close to zero, i.e., $f_{i}<0.5$, the corresponding sample should be classified to Class Zero. Otherwise, if label $f_{i}$ is close to one, i.e., $f_{i}>0.5$, the corresponding sample will be classified to Class One. This is a great option compared to traditional spectral clustering methods in which the cutoff needs considerable human intervention. This advantage makes it much easier for clinicians and biologists to use.

### Multiple-class cases

In practice, the samples may belong to more than two classes. For class discovery cases, the class labels can be obtained by recursively applying our model to classify samples into two groups on each step until some stopping criterion is satisfied. Here we propose an intuitive criterion and name it as the minimum similarity score criterion. Formally, the procedure for class discovery with multiple classes is described as follows:

· **Step 1**: Classify samples into two classes by OTCC.

· **Step 2**: Calculate the inner minimum similarity score for each class. If the minimum similarity score of some class is less than a predefined threshold, then repeat **Step 1** to classify the samples of this class into two sub-classes.

· **Step 3**: repeat **Step 2** until all the inner minimum similarity scores of the classes are above the threshold.

The procedure does not require the number of clusters but instead relies on the least tolerant similarity score within classes. Compared to the number of clusters which is generally required by many existing class discovery methods, our similarity score is tightly related to the expert’s knowledge and is expected to be defined by clinicians and biologists based on their knowledge. Alternatively, without pre-defining a stopping criterion, OTCC can be applied recursively until each sample is a single class. This outputs a binary tree in which all samples are leaves and the relationships among them are fully depicted. This property allows OTCC to reveal the fine structure of patient samples.

For class prediction cases, the relationship between multiple classes can be organized as a binary tree and then the model can be applied recursively according to the binary tree to obtain the labels of all samples. The binary tree should reflect the relationship of the classes. Otherwise wrong prior information will be introduced and mislead the class prediction results. When the class relationships are not available or all the classes are independent of each other, an arbitrary binary tree can be used. One-vs-one or one-vs-all strategies can also be adopted to extend OTCC to multi-class cases.

## Results and discussion

### Performance of OTCC on simulated data sets

**Figure 1:**
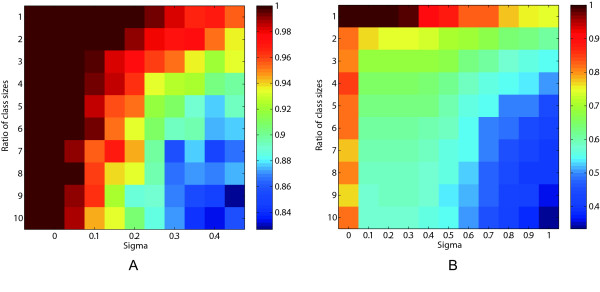
**Clustering accuracy of OTCC (A) and Affinity Propagation (B) on simulated data sets with various levels of noise and ratios of class sizes**. “Sigma” is the standard variation of noise distribution.

We first evaluated OTCC on a simulated dataset and compared the results with those that can be obtained using the existing method. Two types of datasets were simulated. The first dataset consisted of two classes. One class had five samples and the other had n-fold samples relative to the first class. We directly simulated the similarity matrix of the samples. The similarity scores of the two samples from the same class were set to be one and the similarity scores of two samples from different classes were set to be zero. Then noise subjected to a normal distribution with mean zero and standard variation “Sigma” was added. Each setting (noise and ratio of class sizes) was repeated 1000 times. With various levels of noise and ratio of class sizes, the performance of OTCC was noted, and is shown in Figure 1A. It suggests that the accuracy of OTCC does not vary according to the ratio of class sizes when noise in the similarity matrix is low. When noise in the similarity matrix is high, the accuracy of OTCC decreases while the class size ratio increases. The performance of affinity propagation clustering [24] on the same data set was also noted and is shown in Figure 1B. It suggests that OTCC is more robust to noise in the similarity matrix than affinity propagation clustering.

The second simulation dataset consisted of multiple classes and was generated using a similar procedure. For multiple classes, we applied OTCC recursively to construct a binary tree to reveal the multiple classes. If the real relationship among multiple classes is indeed a binary tree, it is reasonable to expect OTCC to succeed. Here we consider an extreme example to show that OTCC can also successfully deal with cases in which the relationship among multiple classes is inherently not a binary tree.

**Figure 2:**
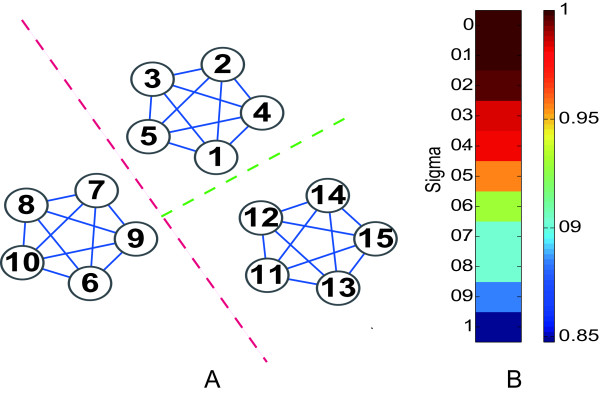
A, a simple simulated data set with three classes; B, performance of OTCC on multiple classes with unbalanced classes and various levels of noise.

In Figure 2A, we demonstrate a graph with three connected components. Each connected component forms a completely connected graph with five nodes. Because the three connected components are equivalent, a binary tree is not the best way to represent their relationships. We constructed a similarity matrix by calculating the Pearson correlation coefficients of the connection vectors of each node pair in the adjacency matrix. The minimal tolerant similarity score is zero and Node 1 and Node 6 are the most dissimilar node pair. OTCC first classifies Nodes 1 to 5 and 11 to 15 as one group and clusters Nodes 6 to 10 as the other group. Because the intra-similarities of the second group all equal to one, i.e., the highest similarity score, there is no cluster structure within the second group. Since the minimal intra-similarities of the first group is still below zero, OTCC is applied again to the first group and distinguishes Nodes 1 to 5 from Nodes 11 to 15 correctly. Calculating the average similarity among the three groups reveals their equivalence.

The success of OTCC for resolving the above multi-cluster structure lies in its ability to form pseudo-clusters when clustering. There are two globally optimum solutions in this case (Nodes 11 to 15 have the same labels as Nodes 1 to 5 or Nodes 6 to 10). OTCC assigns Nodes 11 to 15 to the same labels as Nodes 1 to 5, generating a degenerative pseudo-cluster whereas Nodes 6 to 10 are classified correctly first. We recursively applying OTCC to pseudo-clusters until the consistence criterion applies to each cluster. In this way it resolves the multi-cluster structure irrespective of whether the relationship among the multiple classes is inherently a binary tree or not.

In Figure 2A, the three clusters are balanced (with the same number of nodes). We also simulate the unbalanced and noisy data set by changing the number of nodes within clusters and adding between-cluster links. OTCC can still resolve the underlying multi-cluster structure (Figure 2B).

### Experiments on cancer gene expression data sets

Next we use two real data sets to demonstrate the effectiveness and advantages of our models in both class discovery and class prediction settings. One data set is the gene expression profiling of seventy-two acute leukemia patients [5]. In this data set, twenty-five patients were diagnosed as acute myeloid leukemia (AML) and forty-seven patients were diagnosed as acute lymphoblastic leukemia (ALL). ALL can be further divided into two groups: B cell ALLs and T cell ALLs. Totally the expressions of 6817 genes were profiled by DNA microarrays, which provide systematic information to accurately diagnose patients. The other data set is the gene expression profiling of stromal and epithelial cells of five normal and twenty-eight breast cancer patients, in which the normal samples provide proper controls to highlight the specific gene expression patterns of breast cancer samples [25]. Here we apply our model (1)-(2) to investigate the intrinsic structure of these samples for both class discovery and class prediction to illustrate the advantages of our model.

#### Leukemia data

The raw microarray data contain much noise, so we perform data preprocessing before we construct the similarity matrix and do class discovery and class prediction. We first set a ceiling (16,000) and a floor (100) for the intensities and then filter those genes with max/min≤5or max−min≤500so that the informative genes are retained according to a general procedure and a base 10 logarithmic transformation is applied at the end [26]. Here max and min mean the maximum and minimum gene expression values in all the samples, respectively. Totally there are 3,571 informative genes after the filtration. The clustering accuracy of the various methods in this dataset is summarized in Table 1.

**Table 1:** Clustering accuracy of various methods on leukemia data

**Methods**	**AML vs ALLs**	**AMLs vs B cell ALLs vs T cell ALLs**
OTCC	98%	96%
*k*-means*	98%	71%
Spectral clustering in jClust	97%	85%
Affinity propagation in jClust^	97%	94%
Hierarchical clustering	98%	76%

*k-means was run 1000 times and the accuracy was calculated based on running with the minimal objective function; ^, if affinity propagation generated more than predefined clusters, similar clusters would be merged to calculate the accuracy.

**Figure 3:**
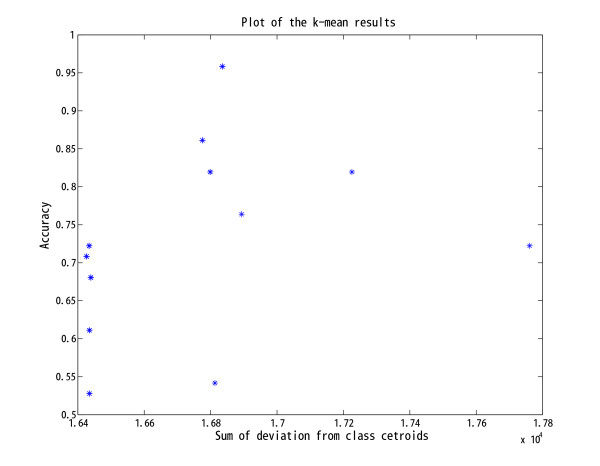
**Clustering accuracy of 1000** ***k*-means runs on the AML and ALL data vs the corresponding objective functions.** The minimal sum of deviation from the class centers (the objective function of *k*-means) does not mean the highest accuracy.

We first applied *k*-means [27,28] (implemented in Matlab 7.11) on this dataset to get a clustering result for reference. K-means tries to identify a center for each cluster and to minimize the sum of deviation of each sample from its corresponding center. Because *k*-means depends on the initial solution and the global optimum is not guaranteed, we ran k-means 1000 times for each dataset and each parameter setting. When k=2*k-*means can correctly discriminate AMLs from ALLs with an accuracy of 98% (71 out of 72 samples correctly classified in 662 out of 1000 runs). However, the accuracy of *k-*means decreases significantly when k=3. It can distinguish AMLs from ALLs (with a poorer accuracy) but it mixes up B-cell ALLs and T-cell ALLs. Only 26 out of 1000 runs achieve more than 90% accuracy but these classifications cannot be selected out by comparing the values of the objective functions (Figure 3). This suggests that *k*-means, the popular tool for clustering, is not effective in revealing subtle subtypes of cancer.

**Figure 4:**
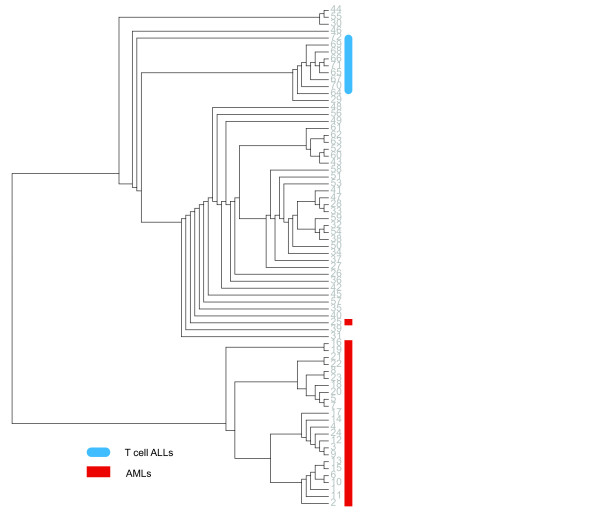
**The classes underlying the seventy-two AML and ALL samples in the leukemia data set revealed by OTCC with the class discovery setting.** Samples 1, · · ·, 25 are AMLs. Samples 26, · · ·, 62 are B cell ALLs. Samples 63, · · ·, 72 are T cell ALLs.

To highlight the pattern underlying the AML and ALL samples, we construct a similarity matrix by first calculating the Pearson correlation coefficients of the gene expression profiles and then calculating the Pearson correlation coefficients of the similarity vectors of each sample. That is, the similarity vectors of each sample (the similarity relationships to other samples) are treated as new features. Then we apply our model (1)-(2) recursively to explore the groups underlying the samples. The result is shown as a rooted tree (Figure 4). The seventy-two samples are first divided into two groups. One group contains twenty-four samples all of them AMLs. The other group contains forty-eight samples which are all ALLs except for sample 25, which is AML. So there is only one sample misclassified (1/72). Subsequent class discovery distinguishes T cell ALLs from B cell ALLs on the fourth clustering in the ALL group. Samples 64, · · ·, 71 and sample 29 are classified as a group, in which all are T cell ALLs except sample 29. Sample 72 (T cell ALL) is recognized as an outlier of the ALL majority. The accuracy reaches 96% (45/47). This observation is consistent with the prior knowledge of this data set, suggesting the effectiveness of our model for class discovery [5].

Applying the spectral clustering to the same similarity matrix (implemented in jClust [29]), the AMLs are grouped correctly except sample 14 and 25. This is similar to our method. But it cannot distinguish B cell ALLs from T cell ALLs (T cell ALLs merged with B cell ALLs completely). Even if changing the input similarity matrix of spectral clustering to the pairwise Pearson correlation coefficients of the gene expression profiles, spectral clustering cannot discriminate AMLs from ALLs.

We also evaluated the affinity propagation algorithm [24] implemented in jClust [29]. The affinity propagation algorithm inputs similarity scores between samples and does not require a predefined number of clusters. We find that our method outperforms jClust in accuracy using the same similarity matrix as our algorithm. In total seven groups are generated by affinity propagation with default parameters. Two groups are AMLs and other groups are ALLs. Sample 25 is misclassified as ALL whereas sample 56 is misclassified as AML. Sample 72 is mis-clustered with B cell ALLs and sample 35 is misclassified as T cell ALLs. Changing the input similarity matrix to the pairwise Pearson correlation coefficients of the gene expression profiles, the affinity propagation algorithm generates three groups, AMLs, B cell ALLs and T cell ALLs, but the accuracy is even lower (61/72 = 84.7%). We also tried different parameter values and the clustering accuracy cannot be further improved.

Agglomerative hierarchical clustering is another popular tool for analyzing the subtle structure underlying the gene expression profiles of cancer samples. Applying agglomerative hierarchical clustering with Euclidean distance to the AMLs and ALLs dataset, it can identify AMLs from ALLs except sample 25. But it failed to discriminate B cell ALLs from T cell ALLs (accuracy: 31/47 = 66%). The T cell ALLs and a set of sixteen B cell ALLs form one cluster whereas other B cell ALLs form the other cluster. The failure of the agglomerative hierarchical clustering for discriminating T cell ALLs from B cell ALLs can be attributed to the fact that the bottom-up cluster merge strategy is a greedy one and cannot find global optimum.

**Figure 5:**
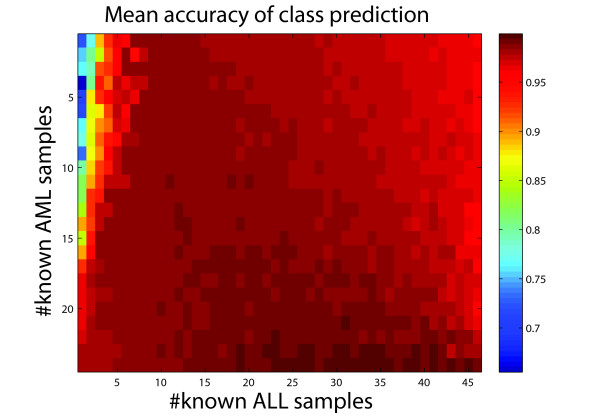
**Mean accuracy heatmap by applying our model to predict the labels of samples in the leukemia data set given labels of certain samples.** Each condition was repeated one thousand times.

Given the known labels of some samples, our model can also carry out the class prediction task. Using the same data set, we evaluate the performance of our model under different conditions in which a fraction of sample labels are known. Given the numbers of each type of samples whose labels are known, we randomly select the same numbers of samples as the prior knowledge and then apply our model to predict the labels of the remaining samples. Repeating one thousand times, we calculate the mean accuracy. The result is shown in Figure 5. It can be seen that the mean accuracy increases with the prior knowledge and that a leap occurs at the initial addition of prior knowledge. This indicates the power of our model to incorporate prior information in a flexible way for class prediction.

#### Breast cancer data

**Figure 6:**
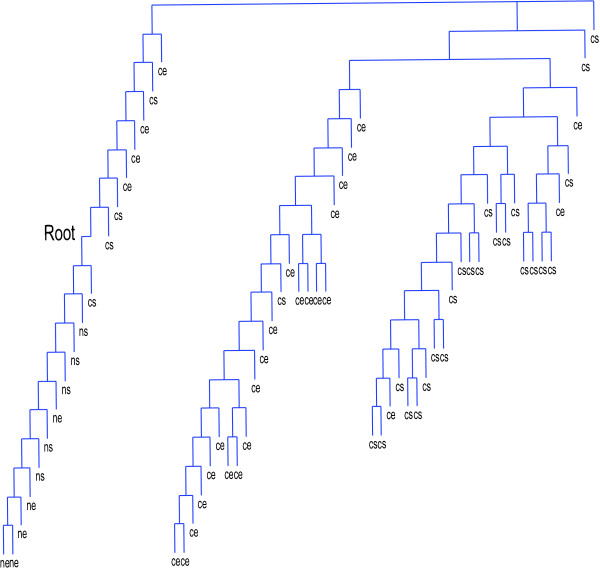
The three major classes underlying the fifty-six breast cancer samples and ten normal samples.

The leukemia data set is assumed to be easy because there are many informative genes which indicate the underlying cluster structure. We repeat the evaluation on another breast cancer dataset to illustrate the advantages of our model on noisier data sets. Since the data set is generated by profiling the gene expressions of stromal and epithelial cells of five normal and twenty-eight breast cancer patients, the samples belong to four classes: normal stromal cells (ns), normal epithelial cells (ne), cancer stromal cells (cs), and cancer epithelial cells (ce) [25]. We apply OTCC to the selected informative genes for both class discovery and class prediction. The top forty-nine genes correlated to normal-cancer discrimination and the top twenty-five genes correlated to stromal-epithelial discrimination (Pearson correlation coefficient > 0.6 or < −0.6) are used as the biomarkers. We calculate the correlations among samples to construct the similarity matrix. Our model for class discovery identifies three major groups: the normal group, the cancer epithelial group and the cancer stromal group (Figure 6). It can be seen that the normal samples are distinguished from the cancer samples. The cancer stromal samples and cancer epithelial samples make independent groups, respectively. But the normal stromal samples do not form a closely-related group. This is different from the original experimental design, implicating the fact that the normal stromal samples may be heterogeneous or the data may contain much noise. Classical spectral clustering reveals the normal-cancer structure but cannot discriminate cancer epithelial cells from cancer stromal cells, or normal epithelial cells from normal stromal cells. The agglomerative hierarchical clustering gets the same result as OTCC.

**Figure 7:**
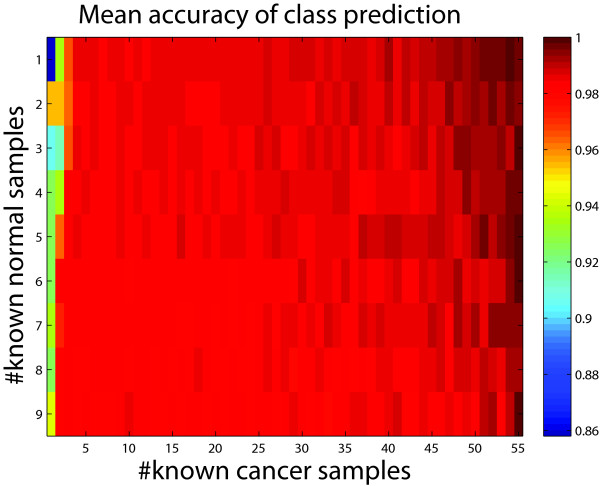
**Mean accuracy heatmap by applying our model to predict the labels of samples in the breast cancer data set given labels of certain samples.** Each condition was repeated one thousand times.

Given some prior information about the labels of the samples, we applied our model to this data set in the class prediction setting. We obtained similar observations to the leukemia dataset (Figure 7), This fact further suggests the advantage of our method in noisy datasets.

### Property summary of OTCC compared to other methods

Gene expression profiling technologies, e.g. microarrays and deep sequencing, have become more and more important for clinical practices, such as diagnosis and prognosis. Class discovery and class prediction are two typical tasks to utilize gene expression profiling technologies to leverage the quality and efficiency of diagnosis and prognosis. In this study, we propose a novel optimization model and integrate two tasks in one framework by treating class discovery and class prediction as a process of labeling. By seeking an optimal labeling scheme that fits best to the gene expression profiling of samples, a convex quadratic programming model is established. It can be solved efficiently and the global optimum solution is guaranteed. It does not need manual intervention to set a cutoff and can detect outliers to improve the statistical signal in the data. It does not use directly the clinical measurement but rather uses a similarity matrix as its input. The biomarker identification process is thus separated from class discovery and class prediction, facilitating clinicians to integrate prior knowledge with the measurements. It can also be applied to multiple types of measurements, e.g. gene expression profiling, proteomic analysis, and next-generation sequencing. Because the similarity matrix is the only input, the output is sensitive to biomarker selection and similarity measures choices. Proper biomarkers and similarity measures will generate reasonable accuracy and greatly accelerate understanding of the nature of diseases. Numerical experiments on leukemia and breast cancer data sets suggest that it is very effective for revealing and predicting the subtle subtypes of cancers based on the gene expression data of patients.

Because the objective function of our model is a quadratic form of the Laplacian matrix, it is closely related to spectral clustering and semi-supervised learning methods. Spectral clustering can be generally solved by seeking the Fiedler vector of the Laplacian matrix [22,26]. The resulting vector sums to zero and the norm equals to one. Because it originates from the matrix eigenvector, it does not provide a natural threshold. So it needs additional selection of cutoffs [22]. Here we model the class discovery and class prediction by explicitly denoting classes by zero and one and then seeking an optimal label assignment to extract the information hiding in the data. A natural cutoff, 0.5, is provided. As opposed to many semi-supervised learning methods in which the unlabeled samples are assigned zero, the positive samples are assigned +1 and the negative samples are assigned −1 [23], we do not assign any labels to the unknown samples, which may prevent artificial bias during modeling. Compared to the frequently used agglomerative hierarchical clustering method, OTCC provides a divisive hierarchical clustering procedure in which the global information is utilized at each step. Compared to *k*-means and fuzzy c-means methods, OTCC can guarantee the global optimum and does not require a predefined number of clusters. This feature is helpful when clinicians do not know how many sub-classes exist for a certain disease. Because it is based on the similarity matrix, it is an open framework that allows prior information to plug in. Numerical experiments on real leukemia and breast cancer data sets suggest the effectiveness of our method, especially its advantage in illustrating the fine cluster structure. Adding partial label information, OTCC turns into a class prediction tool and can reach high accuracy. We note that spectral clustering has also been extended to incorporate constraint information [30,31] for semi-supervised learning. This extends the scope of this study, so the corresponding comparison is not included.

## Conclusions

Class discovery and class prediction are two tasks linked to each other inherently in clinical research. Previous studies proposed methods for these two tasks separately. And thus ignored the linkage between these two tasks. In this study, we model class discovery and class prediction in one framework and facilitate the discovery and prediction of subtle subtypes of cancers. Because of its flexibility, our method can be applied to multiple types of measurements, e.g. gene expression profiling, proteomic analysis, and next-generation sequencing and allows the integration of extensive prior information.

## Abbreviations

HC, hierarchical clustering; SOM, self-organizing maps; NMF, non-negative matrix factorization; OTCC, an Optimization Tool for Clustering and Classification; SMO, sequential minimal optimization algorithm; AML, acute myeloid leukemia; ALL, acute lymphoblastic leukemia.

## Competing interests

The authors declare that they have no competing interests.

## Authors’ contributions

XR proposed the model. YW proposed the fast algorithm. XSZ checked the theoretical properties of the model and the algorithm. XR and JW completed the computation on the datasets. XR, YW, JW and XSZ wrote and approved the manuscript. All authors read and approved the final manuscript.

## References

[B1] Bals R Jany B Identification of disease genes by expression profiling Eur Respir J 2001 18 5 882 889 11757640 10.1183/09031936.01.00106601

[B2] Greenberg SA DNA microarray gene expression analysis technology and its application to neurological disorders Neurology 2001 57 5 755 761 11575306 10.1212/wnl.57.5.755

[B3] Henriksen PA Kotelevtsev Y Application of gene expression profiling to cardiovascular disease Cardiovasc Res 2002 54 1 16 24 12062357 10.1016/s0008-6363(01)00516-8

[B4] Lagraulet A Current Clinical and Pharmaceutical Applications of Microarrays: From Disease Biomarkers Discovery to Automated Diagnostics J Assoc Lab Autom 2010 15 5 405 413

[B5] Golub TR Slonim DK Tamayo P Huard C Gaasenbeek M Mesirov JP Coller H Loh ML Downing JR Caligiuri MA Molecular Classification of Cancer: Class Discovery and Class Prediction by Gene Expression Monitoring Science 1999 286 5439 531 537 10521349 10.1126/science.286.5439.531

[B6] Brunet J-P Tamayo P Golub TR Mesirov JP Metagenes and molecular pattern discovery using matrix factorization Proc Nat Acad Sci USA 2004 101 12 4164 4169 15016911 10.1073/pnas.0308531101 PMC384712

[B7] Gao Y Church G Improving molecular cancer class discovery through sparse non-negative matrix factorization Bioinformatics 2005 21 21 3970 3975 16244221 10.1093/bioinformatics/bti653

[B8] Hsu AL Tang S-L Halgamuge SK An unsupervised hierarchical dynamic self-organizing approach to cancer class discovery and marker gene identification in microarray data Bioinformatics 2003 19 16 2131 2140 14594719 10.1093/bioinformatics/btg296

[B9] Kim H Park H Sparse non-negative matrix factorizations via alternating non-negativity-constrained least squares for microarray data analysis Bioinformatics 2007 23 12 1495 1502 17483501 10.1093/bioinformatics/btm134

[B10] Li W Fan M Xiong M SamCluster: an integrated scheme for automatic discovery of sample classes using gene expression profile Bioinformatics 2003 19 7 811 817 12724290 10.1093/bioinformatics/btg095

[B11] Steinfeld I Navon R Ardigo D Zavaroni I Yakhini Z Clinically driven semi-supervised class discovery in gene expression data Bioinformatics 2008 24 16 i90 i97 18689846 10.1093/bioinformatics/btn279

[B12] Varma S Simon R Iterative class discovery and feature selection using Minimal Spanning Trees BMC Bioinforma 2004 5 126 10.1186/1471-2105-5-126 PMC520744 15355552

[B13] von Heydebreck A Huber W Poustka A Vingron M Identifying splits with clear separation: a new class discovery method for gene expression data Bioinformatics 2001 17 suppl 1 S107 S114 11472999 10.1093/bioinformatics/17.suppl_1.s107

[B14] Yu Z Wong H-S Wang H Graph-based consensus clustering for class discovery from gene expression data Bioinformatics 2007 23 21 2888 2896 17872912 10.1093/bioinformatics/btm463

[B15] Brown MPS Grundy WN Lin D Cristianini N Sugnet CW Furey TS Ares M Haussler D Knowledge-based analysis of microarray gene expression data by using support vector machines ProcNat Acad Sci USA 2000 97 1 262 267 10.1073/pnas.97.1.262 PMC26651 10618406

[B16] Furey TS Cristianini N Duffy N Bednarski DW Schummer M Haussler D Support vector machine classification and validation of cancer tissue samples using microarray expression data Bioinformatics 2000 16 10 906 914 11120680 10.1093/bioinformatics/16.10.906

[B17] Ji Y Tsui K-W Kim K A novel means of using gene clusters in a two-step empirical Bayes method for predicting classes of samples Bioinformatics 2005 21 7 1055 1061 15514000 10.1093/bioinformatics/bti092

[B18] Lee Y Lee C-K Classification of multiple cancer types by multicategory support vector machines using gene expression data Bioinformatics 2003 19 9 1132 1139 12801874 10.1093/bioinformatics/btg102

[B19] Tan AC Naiman DQ Xu L Winslow RL Geman D Simple decision rules for classifying human cancers from gene expression profiles Bioinformatics 2005 21 20 3896 3904 16105897 10.1093/bioinformatics/bti631 PMC1987374

[B20] Alexandridis R Lin S Irwin M Class discovery and classification of tumor samples using mixture modeling of gene expression data}a unified approach Bioinformatics 2004 20 16 2545 2552 15117753 10.1093/bioinformatics/bth281

[B21] Filippone M Camastra F Masulli F Rovetta S Asurvey of kernel and spectral methods for clustering Pattern Recognit 2007 41 176 190

[B22] von Luxburg U A Tutorial on Spectral Clustering Stat Comput 2007 17 395 416

[B23] Hwang T Sicotte H Tian Z Wu B Kocher J-P Wigle DA Kumar V Kuang R Robust and efficient identification of biomarkers by classifying features on graphs Bioinformatics 2008 24 18 2023 2029 18653521 10.1093/bioinformatics/btn383

[B24] Frey BJ Dueck D Clustering by Passing Messages Between Data Points Science 2007 315 5814 972 976 17218491 10.1126/science.1136800

[B25] Casey T Bond J Tighe S Hunter T Lintault L Patel O Eneman J Crocker A White J Tessitore J Molecular signatures suggest a major role for stromal cells in development of invasive breast cancer Breast Cancer Res Treat 2009 114 1 47 62 18373191 10.1007/s10549-008-9982-8

[B26] Kim C Cheon M Kang M Chang I A simple and exact Laplacian clustering of complex networking phenomena: Application to gene expression profiles Proc Nat Acad Sci USA 2008 105 11 4083 4087 18337496 10.1073/pnas.0708598105 PMC2393820

[B27] Macqueen JB Some Methods for classification and analysis of multivariate observations. In: 1967 1967 University of California Press, Berkeley 281 297

[B28] Lloyd S Least squares quantization in PCM Inf Theory, IEEE Trans on 1982 28 2 129 137

[B29] Pavlopoulos GA Moschopoulos CN Hooper SD Schneider R Kossida S jClust: A clustering and visualization toolbox Bioinformatics 2009 25 15 1994 1996 19454618 10.1093/bioinformatics/btp330 PMC2712340

[B30] Yang C Zhang X Jiao L Wang G Self-Tuning Semi-Supervised Spectral Clustering Comput Intell Secur, Int Conf on 2008 1 1 5

[B31] Mishra A Gillies D Bairoch A, Cohen-Boulakia S, Froidevaux C Semi Supervised Spectral Clustering for Regulatory Module Discovery Data Integration in the Life Sciences 2008 Berlin/Heidelberg, Springer-Verlag 192 203 vol. 5109

